# Begutachtung nach dem Opferentschädigungsgesetz (OEG) von Betroffenen sexualisierter Gewalt im Verantwortungsbereich der katholischen Kirche

**DOI:** 10.1007/s00115-023-01588-z

**Published:** 2023-12-29

**Authors:** Raffael Azevedo Antunes Konietzko, Harald Dreßing

**Affiliations:** Zentralinstitut für Seelische Gesundheit Mannheim, Medizinische Fakultät Mannheim, Universität Heidelberg, J 5, 68159 Mannheim, Deutschland

Zunehmend stellen Personen, die schon in fortgeschrittenerem Lebensalter sind und Opfer sexualisierter Gewalt durch katholische Kleriker in ihrer Kindheit oder Jugend geworden sind, einen Antrag nach dem Opferentschädigungsgesetz (OEG). Begutachtungen nach OEG von Betroffenen sexualisierter Gewalt im Verantwortungsbereich der katholischen Kirche bringen besondere Herausforderungen mit sich, da die erlittenen Traumata oft schon Jahrzehnte zurückliegen. Die Autoren haben sich sowohl wissenschaftlich in der Studie „Sexueller Missbrauch an Minderjährigen durch katholische Priester, Diakone und männliche Ordensangehörige im Bereich der Deutschen Bischofskonferenz“ (MHG-Studie) als auch in der praktischen Begutachtung intensiv mit der Thematik befasst und wollen aus diesem Erfahrungshintergrund einige Überlegungen zu dem Thema beisteuern.

## Psychische Störungen nach sexuellem Missbrauch durch katholische Kleriker

Eine Besonderheit ist, dass Betroffene oft mit erheblicher zeitlicher Latenz den erlittenen Missbrauch offenbaren und Entschädigungsansprüche geltend machen.

Dass es erhebliche psychische Folgen bei Betroffenen geben kann, hat die MHG-Studie gezeigt. In der im Rahmen der MHG-Studie durchgeführten Personalaktenanalyse ergab sich, dass bei etwa einem Drittel psychosoziale Folgen in den Akten dokumentiert waren. Ängste (42,4 %) und Depressionen (42,3 %) wurden am häufigsten als Folge genannt. 626 Betroffene (17,0 %) ließen sich in Anbetracht der Tatfolgen psychiatrisch oder psychotherapeutisch behandeln [1]. Mindestens ein Leitsymptom einer Posttraumatischen Belastungsstörung (PTBS) konnte bei 70,2 % der Betroffenen, mit denen ein Interview geführt wurde, festgestellt werden. Bei 44,8 % bestand eine erhebliche Beeinträchtigung der psychischen Situation, die auf einen gegebenen Psychotherapiebedarf hinwies [2]. Dabei ist aber zu berücksichtigen, dass weder durch die Personalaktenanalyse noch durch die biografischen Interviews eine valide psychiatrische Diagnostik erfolgen konnte. Dennoch ist in Kenntnis der oft massiven sexualisierten Gewaltausübung, die von den Betroffenen berichtet wurde, davon auszugehen, dass ein nicht unerheblicher Teil der Betroffenen in der Folge auch psychische Störungen entwickelt hat.

Erhebliche psychische Folgen nach traumatischen Ereignissen sind auch aus anderen Arbeiten bekannt [3]. Ob und in welchem Ausmaß psychische Störungen als Folge des Missbrauchserlebens auftreten, kann aber nur durch eine ausführliche und individuelle Diagnostik erfolgen. Eine solche kann z. B. im Rahmen einer Begutachtung nach dem OEG durchgeführt werden.

## Grundsätzliche Vorgaben bei einer Begutachtung nach OEG

Bei einem OEG-Gutachten kommt es nicht nur auf einen Querschnittsbefund und eine auf dieser Basis erhobene Diagnose an, sondern es müssen auch die Kausalitätsregeln der Begutachtung nach dem OEG beachtet werden, das heißt, das in Rede stehende Trauma muss zumindest als wesentliche Teilursache für eine gegebenenfalls vorhandene psychische Störung festgestellt werden. Wegen der bei diesen Fällen in der Regel immer vorhandenen erheblichen zeitlichen Latenz zwischen Trauma und Begutachtung stellt die Kausalitätsbeurteilung eine besondere Herausforderung dar. Im Laufe eines langen Lebens sind viele möglicherweise konkurrierende Kausalfaktoren wie familiäre Konflikte, berufliche Konflikte, körperliche Erkrankungen und auch traumaunabhängige psychische Komorbiditäten zu berücksichtigen. Gleichzeitig können diese unter Umständen auch mittelbare Traumafolgen sein. Das macht komplexe Überlegungen notwendig.

## Das kirchliche Verfahren zur „Anerkennung des Leids“

Die Mehrzahl der Antragsteller in diesem Kontext hat bereits bei der katholischen Kirche einen Antrag auf die „Anerkennung des Leids“ gestellt. Dieses kirchliche Verfahren wurde mehrfach modifiziert, zuletzt im Januar 2023 [4]. Im Gegensatz zur Begutachtung nach dem OEG findet bei diesem kirchlichen Verfahren keine Begutachtung der Antragsteller statt. Die Anträge werden nach Aktenlage oder nach persönlicher Anhörung durch eine Anerkennungskommission beschieden. Sofern eine Plausibilitätsprüfung positiv verlaufen ist, erhalten Betroffene in der Regel entweder eine finanzielle Anerkennungsleistung oder finanzielle Unterstützung für notwendige Therapien.

Entscheidend für die Anerkennung in dem kirchlichen Verfahren ist dabei in erster Linie nicht der Schweregrad einer gegebenenfalls vorhandenen psychischen Störung oder die im OEG zu berücksichtigenden Kausalitätsüberlegungen, sondern der Schweregrad der erlittenen sexualisierten Gewalt. Es erfolgt letztlich also die Anerkennung des Traumas an sich.

Ausdrücklich spricht die Kirche dabei nur von einer „Anerkennung des Leids“ und nicht von einer Entschädigung. Diese kirchlichen Verfahren werden von vielen Seiten, insbesondere aber von den Betroffenen, als intransparent, traumatisierend und willkürlich kritisiert [5]. In der Zusammenfassung der MHG-Studie findet sich deshalb auch explizit die Empfehlung, diese Prüfungen einer von der Kirche völlig unabhängigen Kommission zu übertragen. Dieser Empfehlung ist die katholische Kirche zumindest bisher nicht gefolgt. Zwar ist die Anerkennungskommission interdisziplinär zusammengesetzt und deren Mitglieder werden von einem mehrheitlich nichtkirchlichen Gremium vorgeschlagen. Letztlich werden die Mitglieder aber von der Deutschen Bischofskonferenz berufen, die auch die Verfahrensregeln beschließt. Ob und gegebenenfalls welche Änderungen im Verfahren zur Anerkennung des Leids die bisher noch nicht rechtskräftige Entscheidung des Landgerichts Köln – bei der einem Betroffenen eine deutlich höhere Entschädigungssumme zuerkannt wurde als in den kirchlichen Anerkennungsverfahren bisher üblich – haben wird, bleibt abzuwarten (LG Köln, Urteil vom 13.06.2023 – 5 O 197/22).

## Gestaltung der gutachtlichen Situation

Sofern Personen, die sexualisierte Gewalt im Verantwortungsbereich der katholischen Kirche erlebt haben, einen Antrag nach OEG stellen, sind sie in der Regel also schon in das kirchliche „Anerkennungsverfahren“ involviert gewesen und kommen zum OEG-Gutachter oft mit erheblichen negativen Vorerfahrungen im Zusammenhang mit der Beurteilung ihres Anliegens durch Dritte. Sie fühlen sich häufig missverstanden, gekränkt, enttäuscht, manchmal auch wütend und berichten über einen als retraumatisierend empfundenen Umgang in dem Anerkennungsverfahren. Gleichzeitig kommen sie zum OEG-Gutachter mit teilweise unrealistischen Erwartungen, dass sie nun zumindest durch eine staatliche Leistung Gerechtigkeit erfahren werden. Dabei besteht bei vielen zu begutachtenden Probanden in diesem Kontext eine erhebliche Unkenntnis über die Vorgaben, die Gutachter bei einer OEG-Begutachtung zu berücksichtigen haben. Deshalb ist es außerordentlich wichtig, dass die vor jedem Gutachten obligate Aufklärung der zu begutachtenden Person über den Inhalt der Beweisfragen und das gutachtliche Vorgehen die oben geschilderte Konstellation besonders sensibel berücksichtigt. Vielen Antragstellern ist nicht bekannt, dass sie im gutachtlichen Kontext zumindest ansatzweise auch noch einmal über das in Kindheit und Jugend erlebte Trauma befragt werden. Es versteht sich von selbst, dass dies äußerst behutsam erfolgen muss. Weiterhin sollten Antragsteller vor Beginn der Begutachtung darüber aufgeklärt werden, dass das in Rede stehende Trauma vom Gutachter keineswegs infrage gestellt wird. Dies wird als Anknüpfungstatsache dem Gutachter ja vorgegeben. Fragen zu möglichen Folgen der stattgehabten Traumatisierung und gegebenenfalls vorhandenen konkurrierenden Kausalfaktoren können von Betroffenen dann u. U. aber dennoch als Infragestellung der erlittenen Traumatisierung fehlinterpretiert werden, selbst wenn die Gutachter dabei sensibel und vorsichtig vorgehen. Auch die grundsätzliche Möglichkeit, dass ein Trauma unzweifelhaft vorliegt, aber eine psychische Störung keineswegs zwangsläufig daraus folgen muss, sollte mit den Probanden vorab besprochen werden. Ein Trauma ist zwar eine notwendige, aber keine hinreichende Bedingung für die Entstehung einer Traumafolgestörung [6, 7]. Auf den diesbezüglichen Unterschied im kirchlichen Anerkennungsverfahren sollte hingewiesen werden. Für Fälle, bei denen ein Missbrauch stattgefunden hat, aber keine gravierende psychische Störung festzustellen ist, bewirkt ein ablehnender OEG-Bescheid u. U. eine erneute Kränkung.

Somit ergibt sich oft eine komplexe Gemengelage. Hierbei ist es gutachtlich wichtig, dass eine transparente und differenzierte Darstellung erfolgt, auch wenn es natürlich das nachvollziehbare Anliegen der Antragsteller ist, alles kausal auf die Traumatisierung im klerikalen Kontext zurückzuführen.

## Ausblick

Ab 01.01.2024 werden im Rahmen des SGB XIV die Regelungen der sozialen Entschädigung grundsätzlich neu gefasst. Bei psychischen Gesundheitsstörungen soll die Wahrscheinlichkeit des ursächlichen Zusammenhangs im Einzelfall vermutet werden, wenn diejenigen medizinischen Tatsachen vorliegen, die nach den Erfahrungen der medizinischen Wissenschaft geeignet sind, einen Ursachenzusammenhang zwischen einem nach Art und Schwere geeigneten schädigenden Ereignis und der gesundheitlichen Schädigungsfolge zu begründen. Diese Neuregelung soll es insbesondere Opfern von in der Kindheit erlebter sexueller Gewalt erleichtern, entsprechende Ansprüche geltend machen zu können, da ein Nachweis der Kausalität in diesen Fällen wegen der langen Latenz bei den bisher geltenden Kausalitätsregeln oft schwierig ist.

Für Begutachtungen in diesem Kontext kann auch die neue Diagnose im ICD 11 komplexe PTBS eine besondere Bedeutung erlangen. Es hat sich gezeigt, dass interpersoneller, prolongierter Missbrauch in der Kindheit einen Risikofaktor für die Entwicklung einer komplexen PTBS darstellt [7, 8]. Dieses Merkmal ist im Rahmen des sexuellen Missbrauchs im klerikalen Kontext häufig erfüllt (Durchschnittsalter zum Zeitpunkt des Missbrauchs 12 Jahre, durchschnittliche Missbrauchsdauer 1,3 Jahre; [1]).
